# Upgrade and downgrade rates of fibroepithelial lesions in a B3 lesion cohort: a 15-year analysis from a university hospital

**DOI:** 10.1093/bjr/tqag105

**Published:** 2026-05-09

**Authors:** Gaurav Jyoti Bansal, Oliver Luton, Anna Powell-Chandler, Lucy Satherley, Elliot Elwood, William Martin, Rhydian Williams, Ffion Evans

**Affiliations:** The Breast Centre, Llandough University Hospital, Cardiff and Vale University Health Board, Cardiff University, Penarth, CF64 2XX, United Kingdom; The Breast Centre, Llandough University Hospital, Cardiff and Vale University Health Board, Penarth, CF64 2XX, United Kingdom; The Breast Centre, Llandough University Hospital, Cardiff and Vale University Health Board, Penarth, CF64 2XX, United Kingdom; The Breast Centre, Llandough University Hospital, Cardiff and Vale University Health Board, Penarth, CF64 2XX, United Kingdom; The Breast Centre, Llandough University Hospital, Cardiff and Vale University Health Board, Penarth, CF64 2XX, United Kingdom; The Breast Centre, Llandough University Hospital, Cardiff and Vale University Health Board, Penarth, CF64 2XX, United Kingdom; The Breast Centre, Llandough University Hospital, Cardiff and Vale University Health Board, Penarth, CF64 2XX, United Kingdom; The Breast Centre, Llandough University Hospital, Cardiff and Vale University Health Board, Penarth, CF64 2XX, United Kingdom

**Keywords:** fibroepithelial lesions (FEL), breast, B3, ultrasound, phyllodes, fibroadenoma

## Abstract

**Background:**

Breast lesions classified as B3 according to UK diagnostic guidelines pose significant management challenges due to their uncertain malignant potential. Within the B3 category, fibroepithelial lesions (FEL) include fibroadenomas (FA), where a phyllodes tumor (PT) cannot be excluded, and benign phyllodes tumors (BPT).

**Objectives:**

To evaluate the upgrade and downgrade rates of B3 classified FA and BPT within a B3 lesion cohort, identify correlations with patient demographics and lesion characteristics, and assess the impact of other variables on diagnostic outcomes.

**Methods:**

Of a total of 332 B3 lesions, a study was conducted on 73 patients with B3-classified FA or BPT diagnosed on core needle biopsy between January 2010 and January 2025. Data on demographics, histological diagnosis, lesion size, imaging findings, procedural data, and subsequent histopathological diagnoses (upgrade or downgrade) were collected. Statistical analyses included multivariate logistic regression. All patients were followed for a median of 8 years.

**Results:**

Of 73 FEL cases studied (36 B3-FA, 37 BPT), the B3-FEL downgrade rate was 37% (27/73), and the FEL upgrade rate was 15% (11/73). Downgraded lesions were associated with a significantly lower mean age (32.17 years vs. 40.65 years in non-downgraded lesions, *P* = .027). Multivariate logistic regression identified lower age as the only predictor for downgrade status. The overall long-term recurrence rate was 2.7%.

**Conclusion:**

Our findings suggest that B3-FELs have high downgrade rates. Younger age is associated with downgrading, while lesion size and ultrasound score are not significant predictors of downgrade. A low long-term recurrence rate of 2.7% was observed.

**Advances in knowledge:**

Given the substantially higher downgrade rates in younger patients and the low long-term recurrence rates for B3-FELs, multidisciplinary teams could refine risk stratification and clinical decision-making.

## Introduction

Breast lesions exhibiting borderline histopathology, classified as B3 lesions according to UK diagnostic guidelines,^1–3^ present a significant clinical challenge. These lesions include fibroepithelial lesions (FEL), encompassing benign phyllodes tumors (BPT), and fibroadenomas (FA) in which a phyllodes tumor (PT) cannot be definitively excluded (B3- FA).^4^

The uncertain malignant potential of these lesions necessitates careful and individualized management decisions. Accurate assessment of upgrade (to malignancy or high-risk lesions) and downgrade (to unequivocally benign) risks is crucial to avoid both under- and over-treatment of affected patients.

Unlike fibroadenomas, phyllodes tumors are less common fibroepithelial neoplasms with variable malignant potential, ranging from benign (BPT) to borderline to malignant. However, both FA and BPT are structurally similar biphasic tumors comprising epithelial and stromal components.

Distinguishing between FA and BPT on a core needle biopsy can be challenging in a small proportion of cases.^4–8^ Our local pathology practice classifies certain fibroadenomas as B3 if phyllodes tumor cannot be excluded. Historically, management of B3 lesions has varied widely, often relying on expert opinion and institutional protocols. Understanding the natural history and predictive factors for the upgrade and downgrade of these lesions is therefore paramount for optimizing patient care. While prior studies have reported varying upgrade rates for B3 lesions,^1^^,^^2^^,^^7^ including B3-FA and phyllodes tumors, there remains a need for comprehensive analyses that incorporate patient demographics, lesion characteristics, and procedural approaches.

In this study, we aimed to evaluate the rates of upgrade and downgrade of B3-FA and BPT within our institution’s B3 lesion cohort over 15 years. Specifically, our objectives were to: (1) determine the overall and lesion-specific upgrade and downgrade rates for B3-FEL; (2) identify correlations between age and lesion size with upgrade and downgrade rates; and (3) assess the impact of other variables on predicting diagnostic outcomes. By providing a detailed analysis of these factors, we aim to inform clinical decision-making for patients with B3 lesions, ultimately improving patient outcomes and reducing unnecessary surgical interventions.

## Methods

### Study design and patient population

This retrospective cohort study was conducted at the University Health Board and included patients diagnosed with B3 lesions on core needle biopsy between January 2010 and January 2025. The study was approved by the local audit department (reference number: Breast/SE/2025-26/03) with a waiver of informed consent and ethical approval due to the retrospective nature of the data collection.

### Inclusion and exclusion criteria

Patients were included if they had a histopathological diagnosis of a B3 lesion on core needle biopsy, in accordance with the UK diagnostic guidelines for breast pathology.^1–3^ The primary lesions of interest for this specific analysis were B3 FA and BPT. Patients were excluded if they had a concurrent or prior history of breast malignancy at the time of B3 lesion diagnosis or if their initial biopsy sample contained invasive carcinoma or ductal carcinoma in situ (DCIS). Eight cases were excluded for inappropriate initial classification after sample cleaning, and 11 cases were excluded for inadequate data.

### Data collection

Data were extracted from the Theatreman Ver2 surgical database and the hospital’s Radiology Information and Picture Archiving and Communication System (PACS) using a standardized data collection form. The following variables were collected for each patient:

- Demographic Data: Age at the time of B3 lesion diagnosis.
- Lesion Characteristics: Histological diagnosis (eg, B3-FA, BPT, papilloma, radial scar, atypical ductal hyperplasia [ADH], lobular carcinoma in situ [LCIS]), lesion size (measured on imaging or pathology reports), presence of atypia on initial and subsequent biopsy/surgical procedure, and mammographic and ultrasound findings (RCR/BI RADS scores,^9^ if available).
- Procedural Data: Type of initial biopsy procedure (core needle biopsy), subsequent surgical excision, and any additional interventions.
- Subsequent Upgrade/Downgrade Data: Histopathological diagnosis from subsequent biopsies or surgical specimens, including documentation of upgrade to malignancy or atypia, or downgrade to benign lesions.
- Malignancy on Follow-up Outcomes: All patients were passively followed for a median of 8 years (1-15 years) by retrospective review of electronic records. Any subsequent breast malignancy, including the date of diagnosis and histopathological characteristics were recorded. Time to malignancy, in months, was calculated as the interval from the initial B3 diagnosis to the date of malignancy diagnosis.

### Definition of upgrade and downgrade

- Upgrade was defined as a subsequent histopathological diagnosis of malignancy (invasive carcinoma or DCIS), high-risk lesion with atypia (atypical ductal hyperplasia or lobular carcinoma in situ) or a borderline/malignant phyllodes on a subsequent biopsy or surgical excision following the initial B3 diagnosis.
- Downgrade was defined as a subsequent histopathological diagnosis of a benign lesion (eg, fibroadenoma without atypia) on surgical excision after the initial B3 diagnosis on core biopsy.

### Statistical analysis

Statistical analysis was performed using SPSS Statistics (Version 29). Descriptive statistics were used to summarize patient demographics and lesion characteristics. Overall upgrade and downgrade rates were calculated. Chi-square or Fisher’s exact tests were used to assess associations between categorical variables (eg, lesion type, procedural approach) and the rates of upgrade and downgrade. Mann-Whitney *U* tests were used to compare continuous variables (eg, age, lesion size) between patients who upgraded, downgraded, or remained stable. Multivariable logistic regression analysis was performed to identify independent predictors of upgrade and downgrade, adjusting for potential confounders. A *P*-value of < .05 was considered statistically significant.

## Results

**Figure 1:**
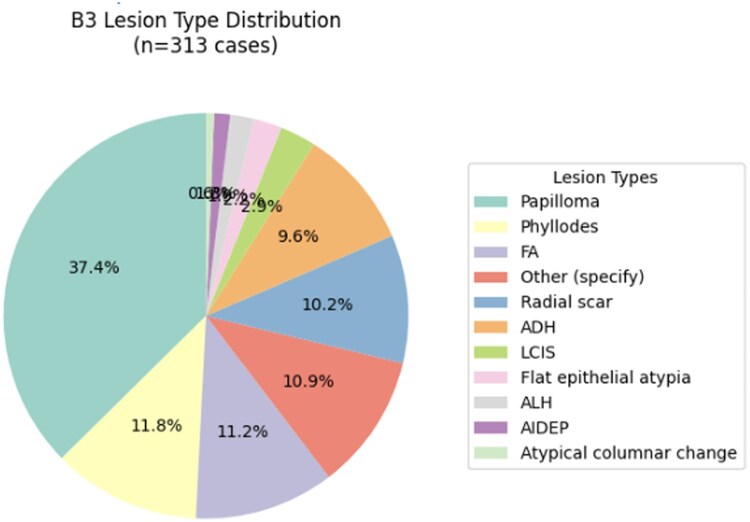
B3 lesion type distribution (*N* = 313 cases); Abbreviations: ALH = Atypical lobular hyperplasia; ADH = Atypical ductal hyperplasia; LCIS = lobular cancer in situ; AIDEP = Atypical intraductal epithelial proliferation. (Please note that FA in the figure refers to B3-FA).

A total of 332 B3 lesions were diagnosed by core needle biopsy between 2010 and 2025. Of these, eight were excluded for concurrent malignancy and 11 for inadequate data. The remaining 313 cases represented the overall B3 lesion cohort. Of this cohort, 73 patients had lesions classified as either B3 fibroadenoma (B3-FA), in which BPT cannot be excluded, or benign phyllodes tumor (BPT), which is the focus of this study. The distribution of B3 lesion types within the entire 313-case cohort is shown in Figure 1. Of the 73 FEL cases analysed, 36 (49.3%) were B3-FA, and 37 (50.7%) were Benign Phyllodes Tumor (BPT).

At our center, serving a population of 500 000, we diagnose approximately 160-200 B2 Fibroadenomas per year across all ages. The B3 FA/BPT rate is approximately 3% (5 per year).

**Figure 2:**
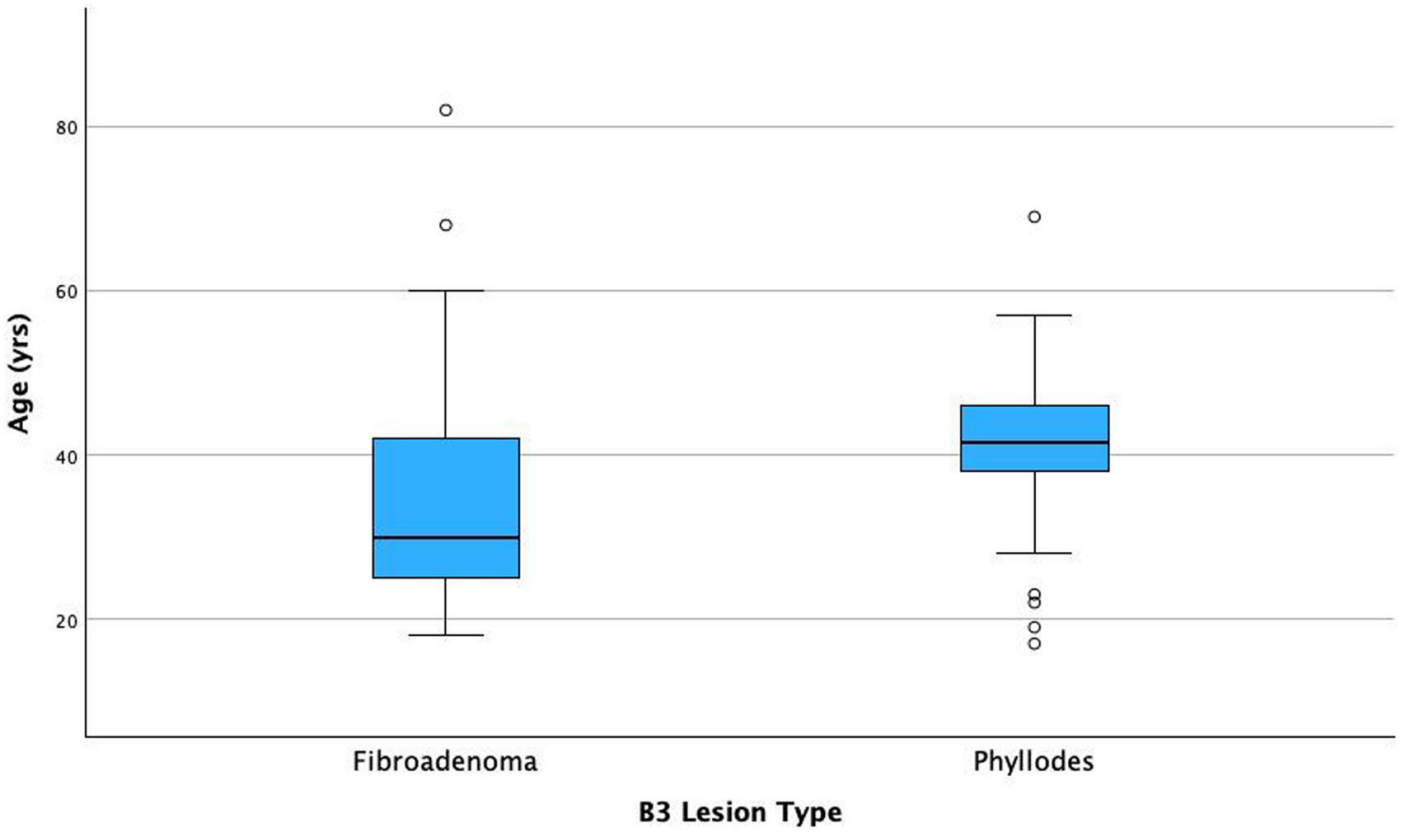
Age distribution by B3 lesion type (Please note that FA in the figure refers to B3 FA).

The median age of the 73 patients with B3 FELs was 30 years (range, 16-82 years) at the time of initial core biopsy. For B3-FA lesions (*N* = 36), the mean age was 35.79 years (range: 18-82 years). For B3-Phyllodes lesions (*N* = 37), the mean age was 41.10 years (range: 17-69 years). The age distribution was negatively skewed (-0.163), indicating a slight tendency towards older ages. Boxplots (Figure 2) indicated that patients with Phyllodes lesions were, on average, older than those with B3-FA lesions; however, the B3- FA group exhibited a wider range and more outliers.

**Figure 3:**
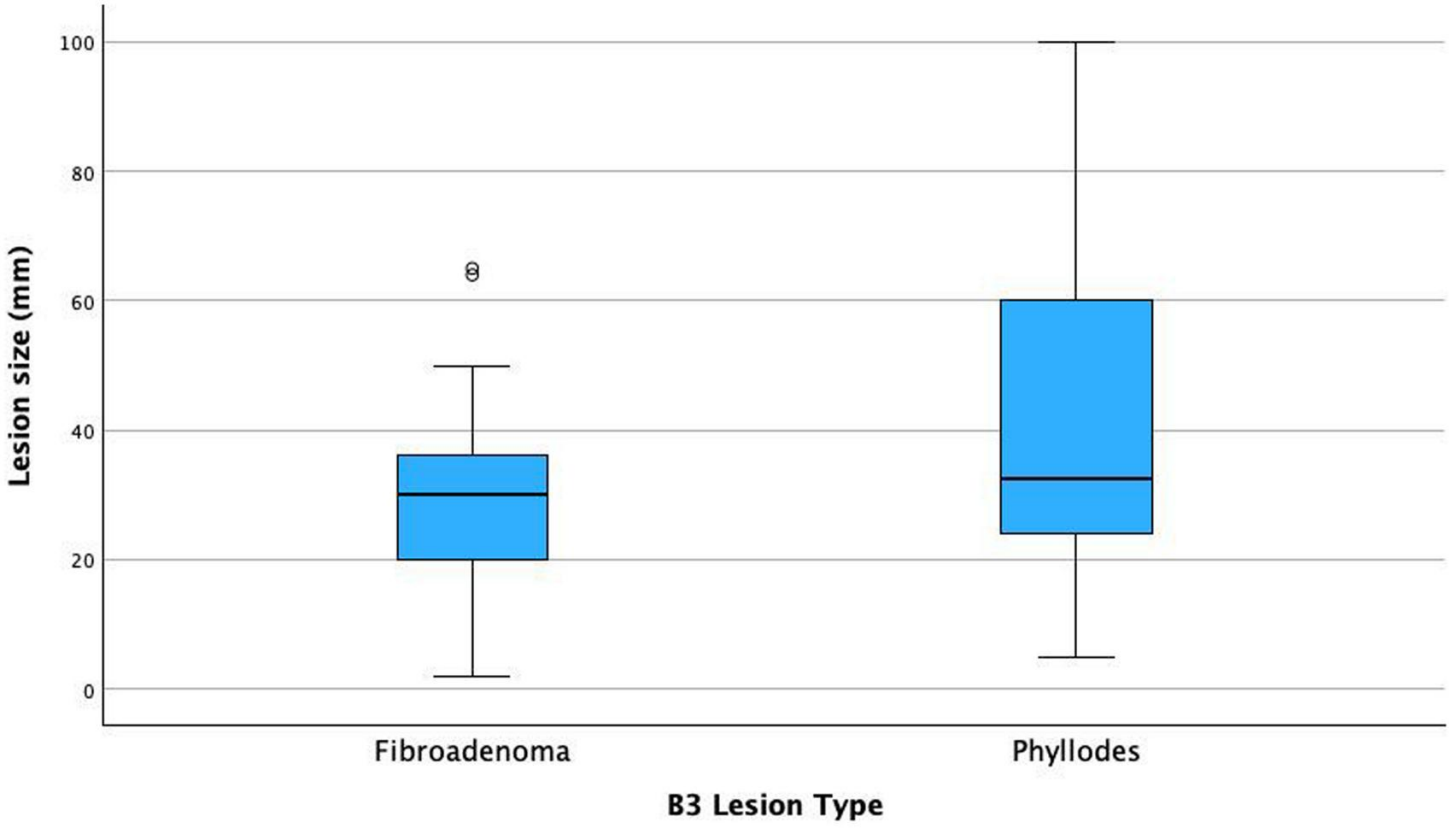
Lesion size distribution by B3 lesion type. (Please note that FA in the figure refers to B3-FA).

The median lesion size for the entire B3-FEL group (*N* = 73) was 32.50 mm (range: 2-100 mm). For B3-FA lesions (*N* = 36), the mean lesion size was 28.76 mm (range: 2-65 mm). For B3 Phyllodes lesions (*N* = 37), the mean lesion size was 42.43 mm (range: 5-100 mm). Boxplots (Figure 3) indicated that Phyllodes lesions were generally larger and had a wider spread than FA lesions, with some outliers observed in the FA group.

**Figure 4:**
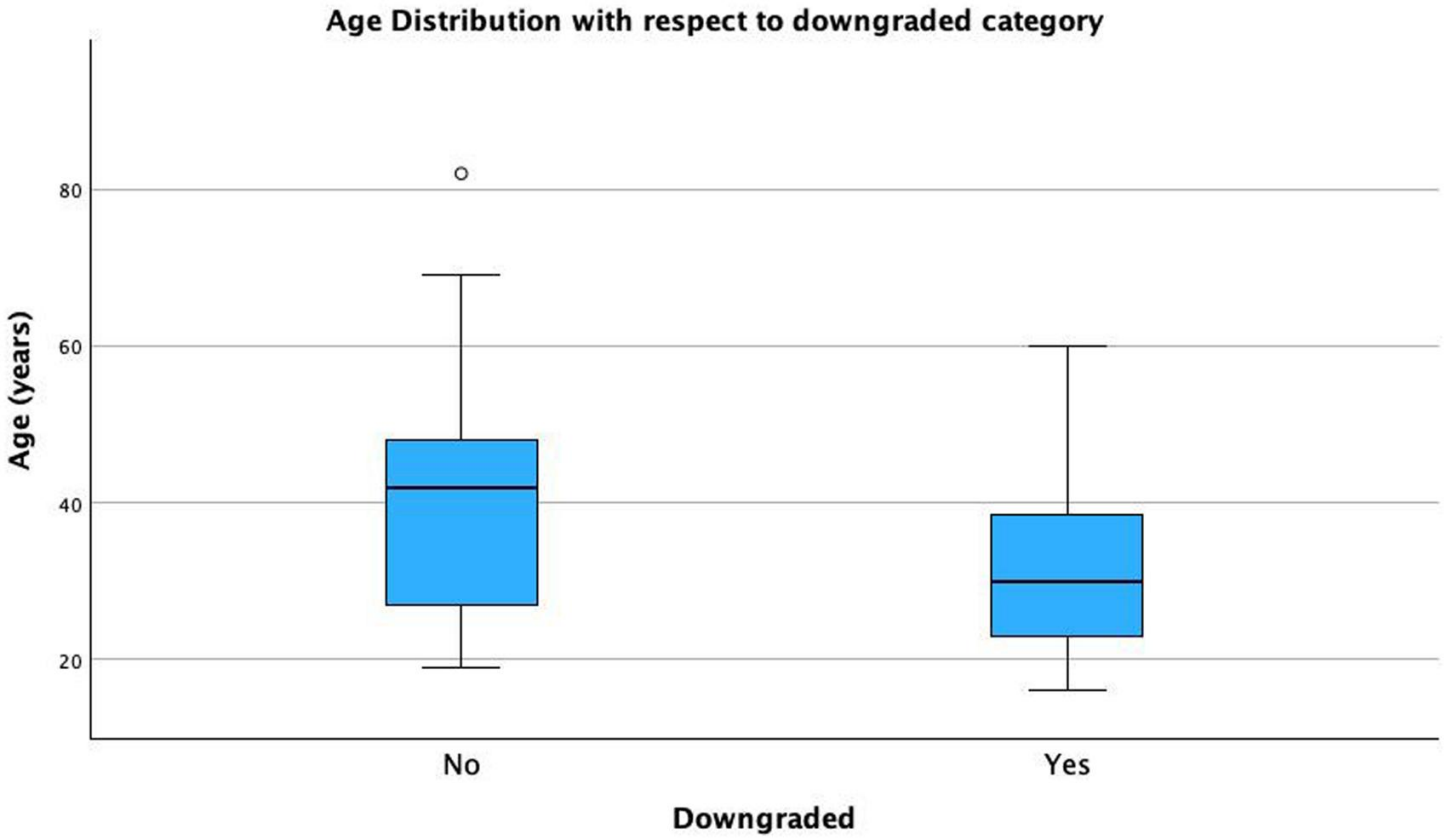
Age distribution with respect to downgraded category. (Please note that FA in the figure refers to B3-FA).

**Figure 5:**
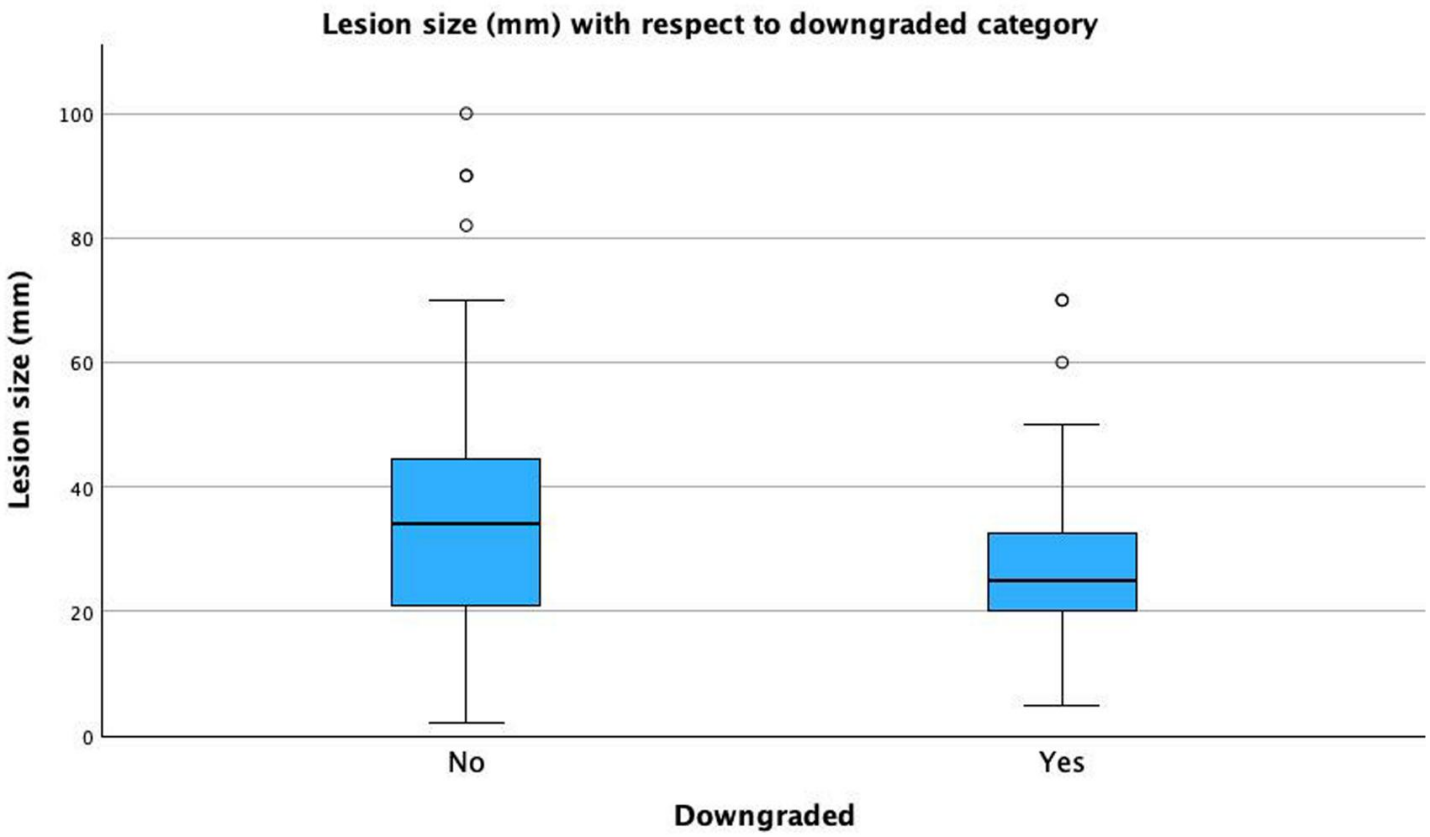
Lesion size distribution with respect to downgraded category. (Please note that FA in the figure refers to B3-FA).

A total of 27 lesions (27/73, 37%) were downgraded to fibroadenoma on subsequent surgical excision. For downgraded lesions (*N* = 27), the mean age was 32.17 years (range: 17-56 years), and the mean lesion size was 29.09 mm (range: 5-70 mm). For lesions not downgraded (*N* = 37), the mean age was 40.65 years (range: 19-82 years), and the mean lesion size was 38.08 mm (range: 2-100 mm). Boxplots of lesion size and age showed that the downgraded group tended to have lower values and narrower distributions than the non-downgraded group (Figures 4 and 5).

Eleven lesions (11/73, 15%) were upgraded on subsequent surgical excision. One B3-FA was upgraded to Borderline PT, and 4 were upgraded to FA with atypical cells following surgical excision. With respect to BPT, 4 and 1 were upgraded to borderline and malignant PT, respectively and one to PT with atypical cells following surgical excision.

**Figure 6:**
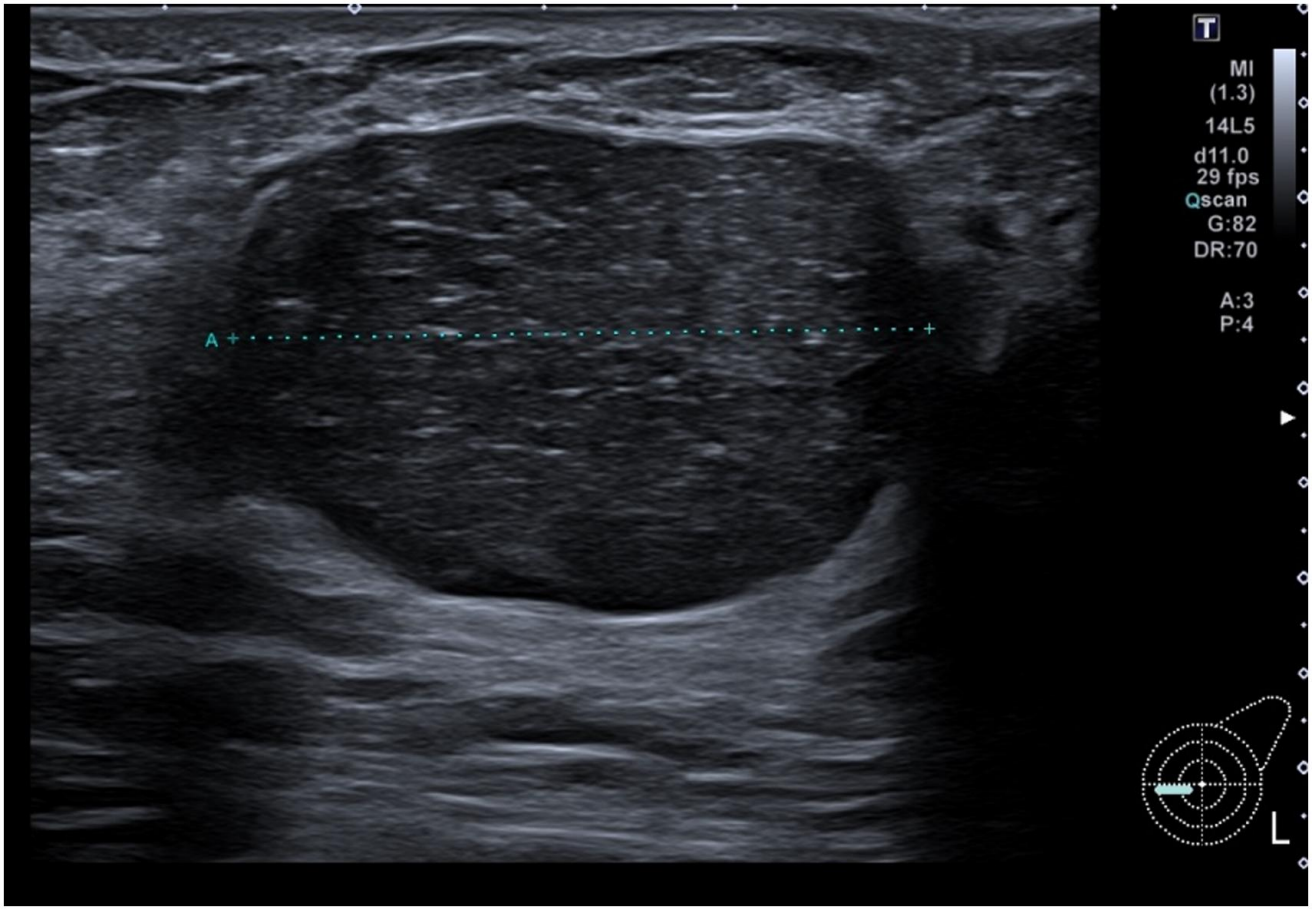
Ultrasound images of an upgraded lesion (B3-FA to intermediate Phyllodes Tumor). Neither size nor ultrasound score was predictive of upgrade.

**Figure 7:**
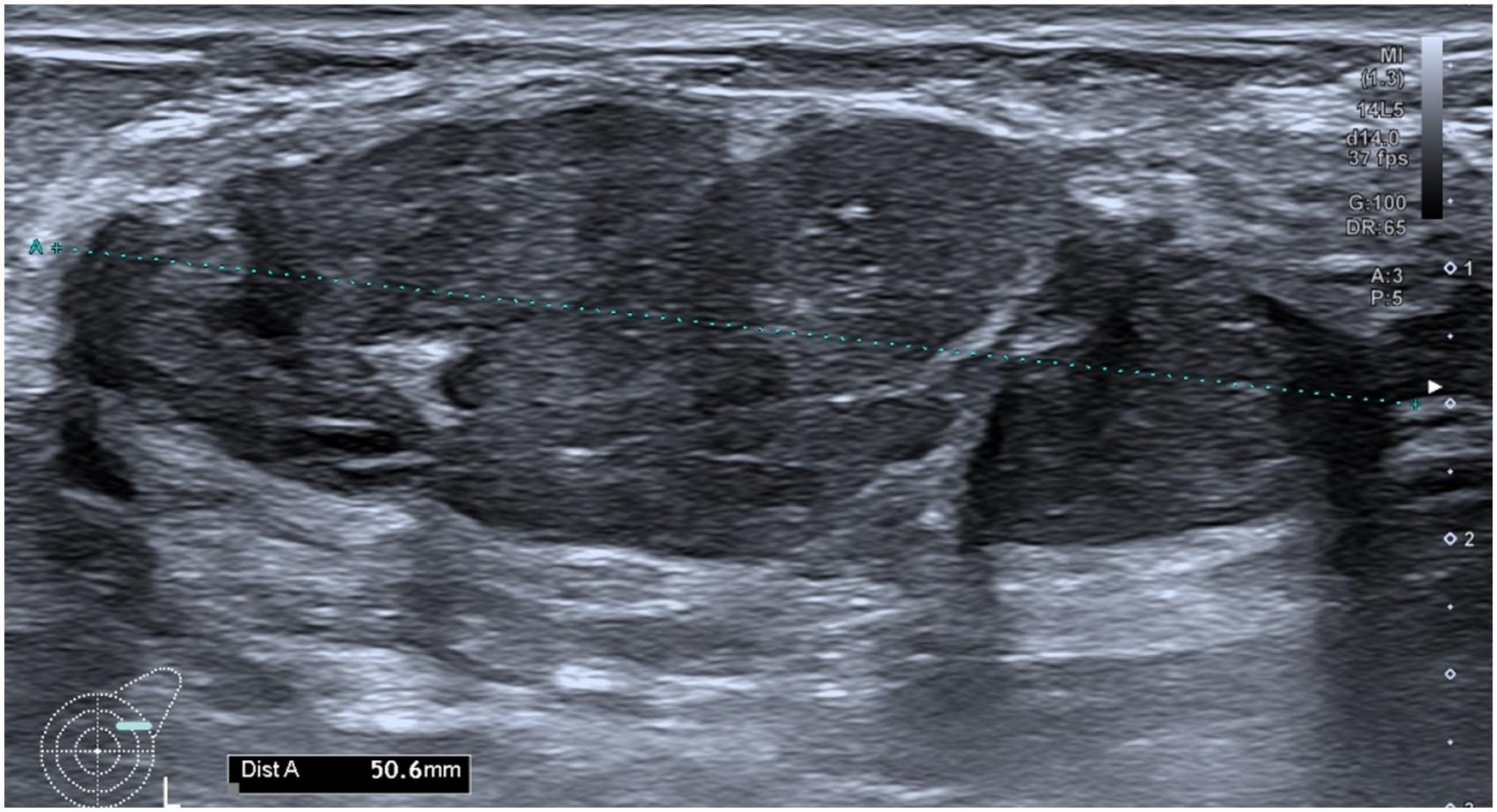
Shows an ultrasound image of a downgraded lesion (BPT to B2 FA). Neither size nor ultrasound score was predictive of downgrade.

- For upgraded lesions (*N* = 11), the mean age was 45.90 years (range: 22-82 years). The mean lesion size was 39.27 mm (range: 2-65 mm).
- For non-upgraded lesions (*N* = 46), the mean age was 36.43 years (range: 17-68 years). The mean lesion size was 33.85 mm (range: 5-100 mm). Figures 6 and 7 show ultrasound images of an upgraded lesion (B3-FA to Bprderline PT) and a downgraded lesion (BPT to FA), respectively.

Most patients (*N* = 65, 89%) underwent image-guided surgical excision. Central/duct excision and vacuum-assisted excision (VAE) were performed in 8 and 2 patients, respectively. VAE was performed in two patients. One was a 14 mm mass (B3-FA), which underwent US-guided VAE, followed by wire-guided excision in 30 year old female. The other was a 55 year old female with a 4 mm cluster of microcalcifications with B3-FA, which was later placed on a B3 pathway (2-yearly mammograms). There was no surgical excision in the last patient, as per the patient’s wishes.

The Pearson Chi-Square test showed no statistically significant association between B3 lesion type (FA or Phyllodes), procedure type, and downgrade or upgrade status [χ^2^(2) = 1.520, *P* = .468].

Nonparametric Tests (Kruskal-Wallis H Test) revealed that the only significant association was observed for age across Downgraded Categories [χ^2^(2) = 6.877, *P* = .032]. Pairwise comparisons (adjusted for Bonferroni) indicated that lesions not downgraded (mean rank 46.10) had significantly higher age ranks compared to downgraded lesions (mean rank 27.67) (*P* = .027). These findings suggest that patients with non-downgraded lesions were older than those with downgraded lesions. The Kruskal-Wallis H test showed no statistically significant difference in lesion size across the downgraded categories [χ^2^(1) = 2.964, *P* = .085].

Multivariable logistic regression analysis to predict upgrade or downgrade status showed no association between lesion size, atypia, multifocality, or imaging score and either upgrade or downgrade status. The only significant finding was that higher age predicted upgrade status, whereas lower age predicted downgrade status (*P* = .03) (Table 1).

**Table 1:** Table showing odds ratio with 95% confidence intervals (95%CI) of factors predicting downgrade of B3 FEL following surgical excision.

Predictor	Odds ratio (OR)	95% CI	*P* value	Interpretation
Age	0.941	0.888-0.998	**.042** ^a^	Increasing age decreases the odds of a downgrade to benign fibroadenoma (statistically significant)
Lesion size at biopsy	0.969	0.931-1.008	.116	Lesion size at biopsy is not a predictor of a downgrade at surgery
Multiple lesions	0.877	0.677-12.10	1.00	Multiple lesions at biopsy are not a predictor of a downgrade at surgery
Ultrasound score	0.041	0.012-14.55	1.00	Ultrasound score at biopsy is not a predictor of a downgrade at surgery
Atypia on first biopsy	0.919	0.61-13.96	.952	Atypia at biopsy is not a predictor of downgrade at surgery

a Significant values are depiected in bold.

**Figure 8:**
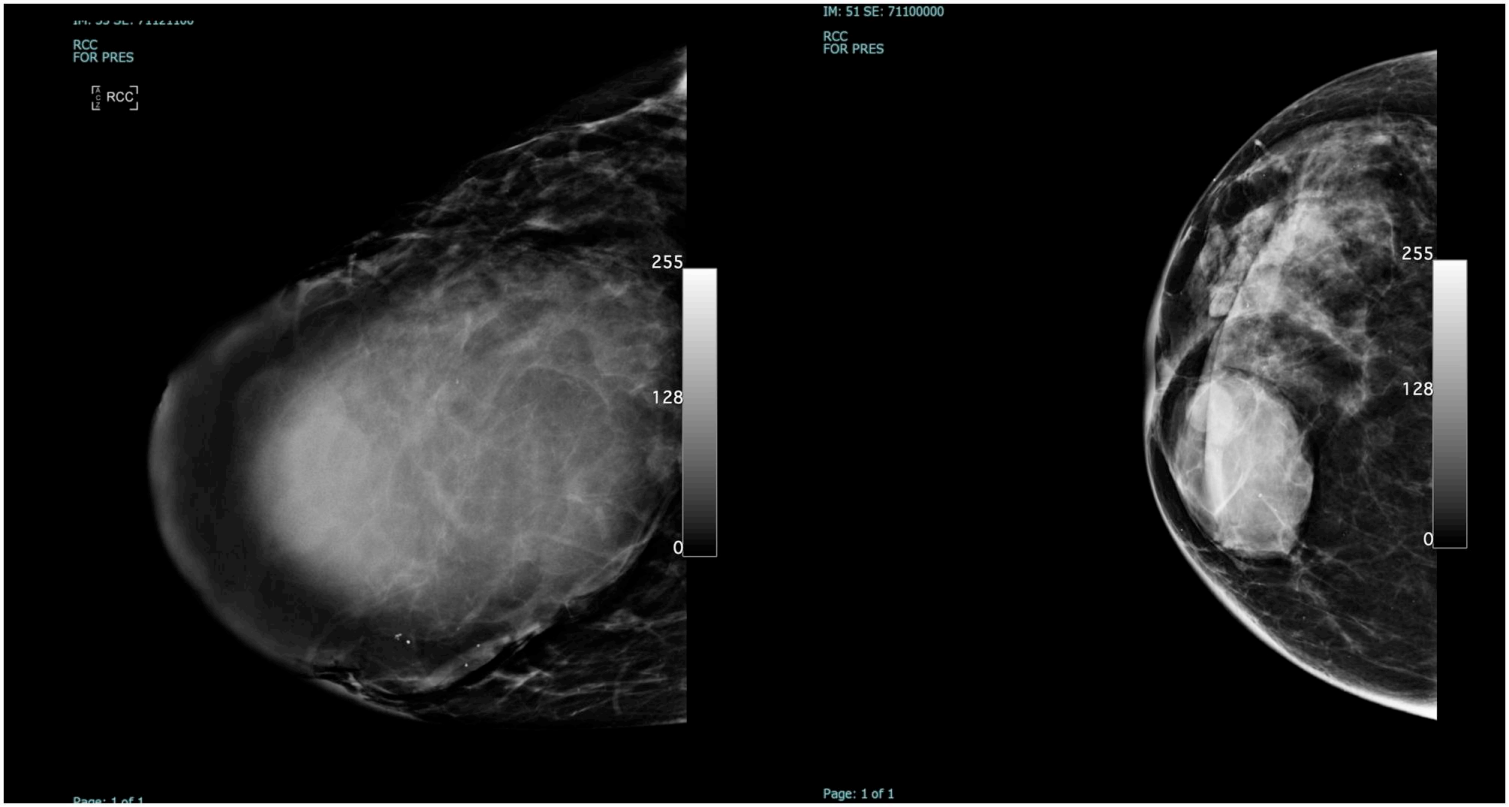
(a, b) 48-Year-old patient whose initial B3-FA (a) was upgraded to a benign and intermediate PT on surgical excision and recurred 8 months later as BPT (b).

Long-term follow-up revealed two cases (2.7% of the 73 FEL cases) with adverse outcomes. The two cases were recurrences of BPT: one was a 48-year-old patient whose initial FA was upgraded to a benign and intermediate PT on surgical excision and recurred 8 months later with a BPT (Figure 8a and b); the other was a 43-year-old patient whose BPT remained BPT on surgical excision and recurred 46 months later as a low-grade BPT. There was also an unrelated case of ipsilateral malignancy in a 43-year-old patient initially diagnosed with BPT, which remained BPT after surgical excision, who developed an incidental Grade 3 invasive ductal carcinoma (IDC) 30 months later at a different site on the same breast.

## Discussion

The management approach of benign breast diseases and proliferative lesions with atypia is limited in evidence and continues to evolve. Despite the worldwide increasing incidence of breast cancers, more women are now being diagnosed with benign breast disease and B3 lesions. This may be due to increased utilization of digital tomosynthesis, greater use of percutaneous core needle biopsies, and improvements in ultrasound technology.^4^

Definitions and management decisions for fibroepithelial lesions (FELs) vary significantly across institutions and remain contentious.^1^^,^^2^^,^^10^^,^^11^^,^^12^ An imaging and clinical concordant breast biopsy is highly accurate in making a diagnosis of benign fibroadenoma with a low false-negative rate. In our center, core biopsies of definitely benign fibroadenomas were labelled as B2 Fibroadenomas. In some cases, differentiating FA from BPT on core biopsies is difficult.^4–8^^,^^13^^,^^14^ This ambiguity arises from the similar biphasic appearance of both tumors on core needle biopsies, each with epithelial and stromal components. Core needle biopsies are particularly challenging due to the small sample size. Moreover, stromal hypercellularity could also be seen in juvenile Fibroadenomas. European guidelines^4^ state that in core needle biopsies, if unable to distinguish between B2 fibroadenoma and Benign or intermediate PT due to stromal hypercellularity, moderate stromal pleomorphism and infrequent mitotic figures, samples should be reported as B3 lesions of uncertain malignant potential. This is in contrast to malignant PT, where diffusely increased stromal cellularity, stromal atypia, and a high mitotic rate (>10/10 high-power field) make it relatively easy to recognize on core needle biopsies.^13^ The diagnostic challenge of differentiating between benign FA and BPT is worth noting, as management differs, although both are indolent lesions not associated with mortality. Typically, FELs with clinical and pathological features concerning for a BPT warrant excision. Moreover, accurate PT classification (benign, borderline, or malignant) generally requires complete excision. In contrast, fibroadenomas without atypia are not routinely excised unless symptomatic and large or increasing in size. Our B3 FEL diagnosis rate of 3% of the total FEL rate is in keeping with previous studies.^5^

Our study’s upgrade rate for FEL lesions, defined as progression to a higher grade at final surgical excision, was 15%. This rate is lower than the reported values in the literature, which range from 25% to 40%.^5–8^^,^^12^^,^^15–17^ Lee et al^7^ retrospective review of 20 cases showed 40% upgrade rate from B3-FEL to malignant or borderline PT on surgical excision. They also found that PT was diagnosed in 12% of cases with a prior core biopsy diagnosis of benign FA, underscoring the diagnostic challenges in differentiating the two on histopathology. However, some studies and expert opinions^2^^,^^3^ suggest substantially higher upgrade rates (up to 75%) when a pathologist strongly suspects PT on core biopsy. This wide variability in reported upgrade rates highlights inconsistent diagnostic criteria, subjectivity, and heterogeneous working practices across centers. Our observed upgrade rate of 15% likely reflects a higher prevalence of benign FAs in our B3 FEL cohort. It suggests a cautious approach by pathologists, employing a low threshold for raising suspicion when encountering equivocal features. Similarly, our FEL downgrade rate was 37%. A higher rate of downgrading further reinforces the validity of local safe practice and the inherent variability in interpretation, even among pathologists within the same center. Our downgrade rate is comparable to that reported in a similar study by Nather et al.,^12^ which included 61 cases and reported a maximum downgrade rate of 38.5%.

Consistent with routine practice and in line with the literature,^4^^,^^6^ most B3 FELs were surgically excised. The rationale for this approach is that BPTs and PTs can continue to grow over time, whereas FAs exhibit growth cessation. Fibroadenomas without atypia and without suspicion of BPT are generally not routinely excised unless symptomatic or reach a certain size.

The optimal margin status for PTs remains a subject of debate.^13–16^ Some studies suggest that a narrow negative margin may be acceptable for FELs suspected of PT or rapid growth, provided the lesion is not transected or morcellated during surgery. While earlier studies, which included benign, borderline, and malignant phyllodes tumors, suggested an association between positive margins and recurrence, a growing body of evidence, particularly for BPTs, indicates that achieving a negative margin may not always be necessary.^2^ Our study specifically included only BPTs within the B3 cohort. We did not evaluate margin status at the time of surgery in our cohort, which is a limitation. However, our observed local long-term recurrence rate was low at 2.7%. Out of the two true recurrences, one was an upgrade from FA to benign/intermediate PT that recurred as BPT at 8 months, and the other was a BPT that recurred as a low-grade BPT at 46 months. These findings align with studies indicating that local recurrence rates can remain low even with positive margins (5-10%).^18–20^

Our analysis revealed that BPT tended to be larger and affected patients slightly older than those with B3-FA. These findings are consistent with other studies^7^^,^^18–21^ showing that PT often presents 1 to 2 decades after FA. Radiologically, both FA and BPT appear similar, with considerable overlap of the imaging features. On Ultrasound, both present as well-defined masses with a gently lobulated contour. Phyllodes tumors are more likely to show cystic spaces and clefts, but these features are present only in a minority of PT.^7^

Importantly, patients who were downgraded were, on average, younger. Although lesion size was not a significant predictor of downgraded status, multivariate logistic regression identified lower age as a significant predictor. Consistent with other studies,^7^^,^^16^^,^^22^^,^^23^ we have not identified absolute criteria for excision of B3 FEL when definitive PT is not evident on CNB, aside from age. This is consistent with previous studies; Lee et al.^7^ found that no combination of clinical, radiological, or pathological features could enable a non-operative diagnosis of all Phylodes tumors.

## Study limitations and strengths

This study has several strengths, primarily its long-term nature (over 10 years) and its B3 cohort focused on FEL. This study also provides unique long-term follow-up data (median of 8 years) on an uncommon entity that is often difficult to obtain. Secondly, this study is unique in that it focused on B3 FELs in young patients rather than on screening age groups, as previous studies have. Thirdly, most patients had surgical excision after initial biopsy as per guidelines. However, certain limitations must be acknowledged. Firstly, a common limitation of retrospective studies with extended passive follow-up periods is that clinical guidelines and practices may have evolved over that period. Moreover, passive follow-up may have missed some recurrences if the person moved out of the area. Secondly, the inherently contentious definitions of FEL and B3 lesions may have led to misclassification of lesions within the study cohort. Thirdly, the margin status of the initial surgical excisions was not evaluated, which limits our ability to assess their impact on recurrence. Fourthly, only traditional ultrasound methods were used to evaluate the ultrasound images, given the long-term historical nature of this analysis.

Given substantially high downgrade rates in younger patients and low long-term recurrence rates for B3 FELs, multidisciplinary teams could refine risk stratification and clinical decision-making. Our findings support the current local practice and published guidelines.^1–3^

We were unable to identify any ultrasound features predictive of a downgrade. In the future, larger-sample studies using advanced ultrasound technologies, including shear-wave imaging and quantitative Doppler vascularity measurements, may be needed to predict the upgrade and downgrade status of B3-FEL and to overcome the limitations of traditional ultrasound methods. Moreover, future advances in immunohistochemistry and molecular characterization may overcome the current diagnostic challenges encountered in FEL diagnosis.

## Conclusion

The definition and management decisions regarding benign fibroepithelial lesions remain variable and contentious. Our study found that phyllodes lesions tended to be larger and affect patients slightly older than those with fibroadenomas. Fibroepithelial lesions had a high rate of downgrade. Downgraded patients were, on average, younger. Lesion size was not a significant predictor of downgraded status. Overall, the long-term recurrence rate remained low at 2.7%. These findings contribute to a better understanding of FEL behavior within a B3 classification, provide insight into current clinical practices, and inform clinical decision-making for patients with these challenging breast lesions.
